# Exploring New Horizons: Advancements in Cartilage Tissue Engineering Under Space Microgravity

**DOI:** 10.7759/cureus.66224

**Published:** 2024-08-05

**Authors:** Madhan Jeyaraman, Swaminathan Ramasubramanian, Sankalp Yadav, Naveen Jeyaraman

**Affiliations:** 1 Clinical Research, Virginia Tech India, Dr MGR Educational and Research Institute, Chennai, IND; 2 Orthopaedics, ACS Medical College and Hospital, Dr MGR Educational and Research Institute, Chennai, IND; 3 Orthopaedics, Government Medical College, Omandurar Government Estate, Chennai, IND; 4 Medicine, Shri Madan Lal Khurana Chest Clinic, New Delhi, IND

**Keywords:** chondrocytes, scaffold design, bioreactor technology, three-dimensional tissue growth, extracellular matrix, regenerative medicine, space microgravity, cartilage tissue engineering

## Abstract

Novel investigations of how microgravity affects cellular and tissue development have recently been made possible by the multidisciplinary fusion of tissue engineering and space science. This review examines the intersection of cartilage tissue engineering (CTE) and space science, focusing on how microgravity affects cartilage development. Space microgravity induces distinct physiological changes in chondrocytes, including a 20-30% increase in cell diameter, a 1.5- to 2-fold increase in proliferation rates, and up to 3-fold increases in chondrogenic markers such as SOX9 and collagen type II. These cellular alterations impact extracellular matrix composition and tissue structure. Space-optimized bioreactors using dynamic culture methods replicate physiological conditions and enhance tissue growth, but the absence of gravity raises concerns about the mechanical properties of engineered cartilage. Key research areas include the role of growth factors in cartilage development under microgravity, biocompatibility and degradation of scaffold materials in space, and in situ experiments on space stations. This review highlights the opportunities and challenges in leveraging microgravity for CTE advancements, emphasizing the need for continued research to harness space environments for therapeutic applications in cartilage regeneration. The multidisciplinary fusion of tissue engineering and space science opens novel avenues for understanding and improving cartilage tissue engineering, with significant implications for the future of biomedical applications in space and on Earth.

## Introduction and background

Cartilage tissue engineering (CTE) stands as a crucial frontier in regenerative medicine, aiming to create viable cartilage tissues for therapeutic applications, particularly in addressing osteoarthritis and cartilage injuries. Traditional approaches on Earth have encountered challenges, including uneven cell distribution and inadequate nutrient diffusion, which have hindered the development of fully functional tissue constructs [1-3]. These limitations stem from Earth's gravity, which influences cell behavior and tissue development, often leading to less-than-ideal structural and functional properties of the engineered tissues [3]. In response to these challenges, space microgravity emerges as a promising alternative, offering a unique environment that significantly alters cellular behavior and tissue development. In microgravity, cells are subject to reduced mechanical stress and different physical interactions, potentially leading to more uniform cell growth and matrix synthesis-key factors in cartilage regeneration [1,4]. The absence of Earth's gravitational constraints in space allows cells to potentially grow in a more natural, three-dimensional manner, potentially closer to how they would in the human body [5].

In microgravity, cells experience profound changes in their behavior and development. Studies have shown that microgravity induces alterations in the cytoskeleton, gene expression, and cellular signaling pathways [6-8]. For instance, mesenchymal stem cells (MSCs) under microgravity conditions exhibit altered differentiation patterns, increased production of reactive oxygen species (ROS), and modifications in stemness-related genes such as Oct-4, SOX2, and Nanog. These changes suggest a complex adaptive response aimed at survival, though they can also lead to premature senescence. The absence of gravitational constraints in microgravity facilitates the formation of more uniform extracellular matrix (ECM) components, resulting in improved matrix synthesis and potentially more functional tissue constructs. Hydrogel-based 3D culture systems in microgravity have demonstrated enhanced cell growth, spreading, and differentiation compared to traditional 2D cultures [9,10]. These 3D systems better replicate the in vivo environment, promoting more physiologically relevant tissue formation. For cartilage tissue engineering specifically, microgravity conditions can mitigate common issues faced in Earth-based approaches, such as uneven cell distribution and inadequate nutrient diffusion. By allowing cells to grow in a more natural, three-dimensional manner, microgravity supports the development of structurally and functionally superior cartilage tissues [4].

This shift towards utilizing space microgravity for CTE is driven not just by academic curiosity but by practical healthcare implications. The exploration of this novel environment could lead to more effective and long-lasting treatments for patients with cartilage-related conditions [4,5,11]. The microgravity environment provides a new platform for overcoming the limitations of Earth-based CTE methods and opens new frontiers in the treatment of joint diseases and injuries, thereby representing a pivotal area of study in modern healthcare [12]. Therefore, the investigation into space microgravity for CTE is more than a scientific endeavor; it's a strategic approach to leverage unique conditions to overcome the limitations faced by traditional Earth-bound techniques. This review examines the potential of space microgravity in enhancing the outcomes of CTE, a field that holds significant promise for treating a myriad of degenerative diseases and injuries afflicting millions globally.

## Review

Microgravity and its effects on cartilage tissue

Microgravity, the state of near-weightlessness experienced in space, significantly influences biological systems by altering cellular behavior, which is not replicable under Earth's gravitational forces. This condition particularly affects cell morphology, proliferation, and differentiation processes. In the realm of cartilage cells, microgravity has been observed to cause enhanced matrix deposition and more uniform tissue development, differing from results seen in Earth-grown counterparts [1,4,13]. Research points to microgravity's role in modulating gene expression pertinent to cartilage development, which is crucial for understanding cartilage growth and regeneration processes [2,14]. In space, the reduction of gravitational force changes cellular behavior, impacting the morphology, proliferation, and differentiation of cells. This environment enables the study of biological processes in ways not possible under Earth's gravitational constraints [2]. Cartilage tissue, vital for joint function and movement, responds distinctly under microgravity. Studies have noted that chondrocytes, the cells responsible for cartilage growth, change shape, size, and function in microgravity. These changes include alterations in gene expression and matrix synthesis, which are essential for cartilage functionality and regeneration [1,14,15].

Microgravity's impact extends to cellular signaling pathways, gene expression, and ECM production, leading to variations in the quality and properties of engineered cartilage. For example, microgravity can enhance the production of specific collagen types, a crucial cartilage component, potentially improving tissue-engineered cartilage quality [3]. The altered mechanical forces in microgravity significantly influence cell behavior and tissue development. Research findings have demonstrated notable effects on chondrocyte proliferation, differentiation, and ECM synthesis, all of which are pivotal elements in the process of cartilage formation and regeneration [12]. These studies have further indicated discernible alterations in gene expression patterns associated with cartilage development and maintenance in the microgravity setting. These genetic changes hold the potential to yield cartilage tissue that closely resembles native cartilage in both structure and function, aligning with a central objective in the field of tissue engineering [1]. Therefore, the unique microgravity environment offers a promising avenue for addressing certain limitations inherent to conventional Earth-based methods of CTE. The comparison of cellular morphology between microgravity and earth-based conditions are tabulated in Table 1.

**Table 1:** Aspects of microgravity conditions and earth-based controls

Aspect	Microgravity Conditions	Earth-based Controls
Cell Morphology	Altered shape and size; more natural cell growth	Standard shape and size typical of Earth-grown cells
Cell Proliferation	Enhanced proliferation rates	Normal proliferation rates
Cell Differentiation	Diversified differentiation pathways, beneficial for cartilage formation	Standard differentiation pathways
Extracellular Matrix Production	Increased synthesis of key components; improved tissue quality	Normal matrix production
Gene Expression	Modulated expression related to cartilage development and regeneration	Standard gene expression patterns
Tissue Architecture	Improved structural and functional properties; more uniform tissue development	Standard tissue architecture; may have non-uniform development
3D Growth	Cells grow in three dimensions, allowing more natural cell-to-cell interactions	Often limited to 2D cultures or less natural 3D environments
Mechanical Properties	Potentially enhanced due to altered response to mechanical stress	Standard mechanical properties
Protein Synthesis	Stimulated synthesis of proteins crucial for cartilage health	Normal protein synthesis rates
Nutrient and Waste Exchange	More efficient due to lack of sedimentation and convection	Can be limited by gravity-induced constraints
Overall Tissue Quality	Closer resemblance to native cartilage in structure and function	May have limitations in mimicking native cartilage

In microgravity, cells grow in three dimensions, allowing more natural cell-to-cell interactions and ECM formations. This environment more closely mimics natural cartilage growth conditions compared to traditional two-dimensional cultures [1]. Under microgravity, chondrocytes exhibit changes in proliferation, differentiation, and ECM production, critical for effective cartilage regeneration [5]. Furthermore, microgravity affects gene expression related to cartilage development at a molecular level, offering new insights for tissue engineering applications [12]. The potential of using space-based platforms for advancing CTE is underscored by microgravity's effects on cartilage tissue. Understanding and harnessing these effects could lead to breakthroughs in treating joint diseases and injuries [1,7]. Microgravity alters various cellular and physiological functions, pivotal in understanding cartilage tissue behavior in space [16,17].

Under microgravity, physical forces acting on cells and tissues differ significantly from Earth. This state leads to substantial changes in cell morphology, proliferation, and differentiation, crucial for cartilage development. The absence of sedimentation and convection in microgravity enables cells to grow in a three-dimensional space, more accurately mimicking the in vivo environment than two-dimensional growth on Earth [4]. Microgravity influences various cell types used in CTE, such as stem cells and chondrocytes. These changes encompass alterations in gene expression, signaling pathways, and cellular metabolism, impacting ECM synthesis, vital for cartilage's structural integrity and function [4,18]. The overall tissue development process is also affected by microgravity, offering a unique perspective on engineered tissue assembly and maturation. Enhanced tissue formation and improved cellular organization have been observed in microgravity, promising for CTE [4].

Devices like the random positioning machine (RPM), clinostat, and NASA's rotating wall vessel (RWV) bioreactor, which simulate microgravity conditions, have been instrumental in advancing our understanding of reduced gravity's effects on tissue development. These devices have provided valuable data on the potential of microgravity to improve CTE outcomes, opening avenues for novel therapeutic applications [4,18,19]. Microgravity provides a unique opportunity to overcome some of the limitations of traditional, gravity-bound tissue engineering methods for cartilage. It allows for a deeper understanding of cellular behavior and tissue development under altered gravity conditions, critical for advancing the field of regenerative medicine. The insights gained from studying cells and tissues in microgravity conditions, as outlined in various studies [1-5,12], are invaluable. They highlight the potential benefits of microgravity in improving the outcomes of CTE.

The scalability and cost-effectiveness of space-based research present significant challenges that need to be addressed for microgravity-based CTE to become a viable option for widespread clinical application. The limited capacity for experiments on space stations and the high costs associated with space missions make it difficult to produce cartilage tissues at the scale needed for clinical use [20-22]. To address this, researchers are exploring Earth-based alternatives such as rotary cell culture systems and random positioning machines that can simulate microgravity conditions [23,24]. These systems, while not perfect replicas of the space environment, offer more accessible and cost-effective platforms for initial research and scaling up production. Additionally, advancements in 3D bioprinting technologies could potentially allow for the rapid production of larger, more complex cartilage constructs once the optimal microgravity-inspired conditions are established [25]. Future efforts should focus on refining these Earth-based microgravity simulation techniques and developing strategies to translate small-scale space experiments into larger-scale Earth-based production methods. This knowledge could lead to significant advancements in developing strategies for cartilage regeneration, offering new possibilities in the treatment of joint diseases and injuries. Therefore, the exploration of microgravity's effects on biological systems, particularly in the context of CTE, is an area of great scientific interest and potential (Table 2).

**Table 2:** Effects of space microgravity on CTE CTE: Cartilage tissue engineering

Aspect of CTE	Impact of Space Microgravity
Cell Morphology	Altered shape and size, leading to more natural cell growth
Cell Proliferation	Enhanced proliferation rates under microgravity conditions
Cell Differentiation	Diversified differentiation pathways, beneficial for cartilage formation
Extracellular Matrix Production	Increased synthesis of key components, improving tissue quality
Gene Expression	Modulated expression related to cartilage development and regeneration
Tissue Architecture	Improved structural and functional properties of engineered cartilage

Current state of CTE

CTE is an essential component of regenerative medicine, aiming to replicate the biomechanical and biochemical characteristics of natural cartilage. This field utilizes a variety of scaffolds, growth factors, and cell types to foster cartilage regeneration. Despite these efforts, replicating the complex structure and functionality of natural cartilage remains challenging, often resulting in tissues with insufficient mechanical strength and inconsistent quality [1]. A significant hurdle in this domain is the influence of Earth's gravity. It affects cell alignment, distribution, and the overall architecture of the engineered tissue, which can lead to tissues that do not possess the structural integrity and functionality required for clinical applications. The mechanical forces of gravity also complicate maintaining the desired cell phenotype and achieving uniform cell distribution within scaffolds [2,26].

These gravity-induced constraints, such as non-homogenous cell distribution and inefficient nutrient transport within tissue constructs, underscore the need for innovative approaches in CTE. Exploring alternative environments like microgravity could potentially overcome these challenges. Microgravity offers a unique setting to study and manipulate cartilage tissue development, which could lead to more effective and reliable engineering methods [3]. Despite the advancements made in CTE, including the development of 3D bioprinting techniques and various scaffolding materials, gravity-dependent limitations persist. These include difficulties in replicating the native cartilage environment and developing scaffolds that mimic natural cartilage's biomechanical properties. Earth-based methods often face issues with insufficient nutrient transport and inadequate mechanical stimulation, critical for the growth and maturation of engineered cartilage [5,27].

Traditional methods in CTE, while advanced, still struggle with replicating the complex structure and biomechanical properties of native cartilage. Challenges like inadequate cell differentiation, limited ECM production, and achieving three-dimensional tissue structures are common. These issues are exacerbated by Earth-based bioreactors' limitations due to gravity, leading to suboptimal cell distribution and tissue formation [4]. In response to these challenges, the exploration of microgravity conditions, as conducted in space-based experiments and simulated through devices like the RPM and RWV bioreactors, is gaining attention. This novel approach holds the potential to bypass the limitations of traditional CTE, offering new possibilities for creating physiologically relevant and functional cartilage tissues. The unique environment of microgravity could be key to addressing the challenges faced in conventional CTE [28].

In CTE, scaffolding materials are crucial for providing a temporary framework that supports cell attachment, proliferation, and differentiation, ultimately aiding in the regeneration of cartilage. Among these materials, natural polymers are widely used due to their biocompatibility and ability to mimic the ECM of native tissues. Table 3 discussed various natural polymers utilized in CTE, their advantages, and limitations.

**Table 3:** Various natural polymers utilized in CTE, their advantages, and limitations CTE: Cartilage tissue engineering

Natural Polymer	Advantages	Limitations
Collagen	Promotes cell adhesion and proliferation, biocompatible, biodegradable	Low mechanical strength, potential immunogenicity
Hyaluronic Acid (HA)	Supports cell migration and proliferation, can be chemically modified	Poor mechanical strength, rapid degradation
Chitosan	Biocompatible, biodegradable, antibacterial properties	Inferior mechanical properties, pH-dependent solubility
Gelatin	Promotes cell adhesion and proliferation, easy to process	Limited mechanical strength, rapid degradation
Alginate	Biocompatible, effective cell encapsulation	Poor mechanical properties, uncontrolled degradation

Research in space microgravity

The exploration of microgravity's effects on biological systems has evolved significantly over time, marked by key milestones and pivotal studies. Initial research began with the advent of human spaceflight in the mid-20th century. The launch of Sputnik by the Soviet Union in 1957, followed by human space missions, prompted the scientific community to study the unique conditions of space, including microgravity [29]. In the 1960s and 1970s, early experiments were conducted aboard spacecraft like the Soviet Vostok and American Mercury and Apollo missions, focusing on basic physiological responses to microgravity [30]. This period laid the groundwork for understanding how the absence of gravity affects the human body, including muscle atrophy and bone density loss. Significant advancements were made with the establishment of space stations. The Soviet Union's Salyut and Mir stations, along with NASA's Skylab, provided platforms for more extended studies. These missions allowed researchers to observe the long-term effects of microgravity on human physiology, plant growth, and microbial behavior. The 1980s and 1990s saw the expansion of microgravity research with the Space Shuttle program, which facilitated numerous scientific experiments in low Earth orbit. During this period, studies highlighted the impact of microgravity on cellular processes, immune function, and the aging process​ [31,32]. In the 21st century, the International Space Station (ISS) has become the primary laboratory for microgravity research. The ISS enables comprehensive studies on various biological systems over prolonged periods. Research has explored areas such as gene expression changes in muscle tissue, the effects on immune cells like dendritic cells, and the interactions between microgravity and space radiation​ [33-35].

Space-based research has significantly advanced our understanding of CTE, a key area in regenerative medicine. Experiments conducted on the Mir Space Station and through ground-based microgravity simulators have revealed notable differences in the morphology and mechanical properties of cartilage tissues developed in space compared to those on Earth. These differences are crucial for grasping the potential benefits and limitations of space-based tissue engineering efforts [1,3]. In particular, a landmark study on the International Space Station, involving the culturing of chondrocytes in microgravity, showcased significant variations in cell morphology and matrix composition when contrasted with Earth-grown counterparts. The results pointed to enhanced chondrogenesis, or the formation of cartilage, under microgravity conditions [2]. This phenomenon indicates that microgravity might be leveraged to create superior cartilage tissues, possessing enhanced structural and functional properties than those achievable on Earth. Such advancements hold promise for more effective treatments for cartilage-related injuries and diseases [3].

Further investigations conducted aboard the International Space Station have delved into the impact of reduced gravity on chondrocyte morphology, gene expression, and ECM production, all of which are pivotal factors in the formation of cartilage tissue [1,36,37]. A noteworthy observation stemming from these experiments pertains to the altered response of chondrocytes to mechanical stress in the microgravity environment, implying the potential for enhancing the biomechanical properties of cartilage tissue. Moreover, microgravity has been noted to stimulate the synthesis of proteins that play a critical role in maintaining cartilage health, thereby suggesting that space environments may hold promise for advancing more effective techniques for cartilage repair [5].

The prevailing techniques in CTE mainly aim to replicate the biomechanical and biochemical properties of native cartilage. However, these methods encounter limitations, notably due to Earth's gravitational forces, which affect cell alignment, differentiation, and ECM formation, all critical for creating functional cartilage tissue [12]. Traditional approaches use scaffolds for structural support and bioreactors for controlled cultivation, but they often fall short in mimicking the complex mechanical environment of natural cartilage. Moreover, the process of converting stem cells into chondrocytes, the primary cells in cartilage, is not entirely efficient under Earth's gravity, leading to inconsistencies in the engineered tissue's quality and functionality [1,5]. These challenges underscore the potential advantages of conducting CTE in space microgravity, where the absence of significant gravitational forces could mitigate many current obstacles.

In the realm of microgravity, researchers have explored the impact of this unique environment on the proliferation and differentiation of chondrocytes. Space-based laboratory studies have reported changes in gene expression and cellular morphology, essential for understanding cartilage regeneration. These findings suggest that microgravity may enhance the chondrogenic potential of cells, a key factor in developing functional cartilage tissue [5,12]. Moreover, the use of microgravity bioreactors has yielded promising improvements in the uniformity and quality of engineered cartilage, potentially addressing some limitations faced by Earth-based bioreactors [1]. Recent studies continue to refine our understanding of microgravity's impact, using advanced technologies like high-throughput sequencing and microarray analysis. These investigations are crucial for developing countermeasures to protect astronauts' health on long-duration missions and for enhancing our knowledge of fundamental biological processes affected by microgravity​ [31,34].

Research comparing space-grown cartilage tissues with those grown on Earth reveals several key differences in morphology and mechanical properties. In microgravity, chondrocytes exhibit more uniform and spherical cell shapes compared to the more elongated and spread-out morphology observed in Earth-grown tissues. This is likely due to the lack of gravitational forces, which affects cellular alignment and distribution within the matrix. Mechanically, space-grown cartilage often demonstrates enhanced functional properties. These tissues show increased ECM production, with higher levels of collagen type II and proteoglycans, contributing to improved compressive strength and elasticity. The unique microgravity environment promotes a more homogeneous tissue structure, which translates into superior mechanical resilience and uniformity in engineered cartilage constructs. These findings are critical for advancing cartilage tissue engineering, suggesting that microgravity conditions can enhance the quality and functionality of bioengineered cartilage, addressing limitations encountered in traditional Earth-based bioreactor systems​ [38]​.

Further space-based research has involved using magnetic fields to manipulate cells in the construction of cartilage tissue, bypassing the need for traditional scaffolding. This approach, conducted in space, has proven the feasibility of tissue engineering in a microgravity environment, underscoring the potential to surpass Earth-bound method limitations. Additionally, devices like the RPM, clinostat, and RWV bioreactor have been employed to simulate microgravity conditions, offering valuable insights into cellular behavior and tissue development under reduced gravity. These studies enhance our understanding of tissue formation and maturation mechanisms in space, crucial for future experiments and for developing new techniques for CTE in microgravity environments [4,28]. This extensive research in space microgravity not only furthers our knowledge in tissue engineering but also opens new pathways for practical applications. The insights gained from these studies are vital for informing future experiments and the development of innovative approaches for CTE in microgravity environments, marking a significant stride in the field of regenerative medicine.

Advancements in microgravity-based techniques

Recent strides in this field include specialized bioreactors designed for microgravity, critical in overcoming traditional limitations related to nutrient diffusion and mechanical stimulation. These bioreactors provide an environment that closely resembles microgravity conditions, essential for cartilage tissue growth [12,39-41]. Advancements in scaffold design have led to materials that support cell growth and facilitate proper cell organization, crucial for functional cartilage tissue formation [1]. Microgravity bioreactors have played a crucial role in enabling three-dimensional growth of cartilage tissues, exhibiting improved cell proliferation and matrix formation compared to Earth-based counterparts [12]. The evolution of scaffold technology has supported cell growth and proper organization, essential for functional cartilage tissue [1]. Progress in cell culture techniques under microgravity has deepened understanding of cellular behaviors in a three-dimensional context, leading to more effective strategies for cultivating cartilage tissues [5]. Tools like the RWV, RPM, and clinostat simulate microgravity, advancing tissue development by promoting natural cell interactions [4]. A groundbreaking development is the use of scaffold-free techniques, where magnetic fields guide cell assembly, enabling formation of more natural cartilage tissues in microgravity [28]. These advancements improve engineered cartilage quality and open new opportunities in regenerative medicine, particularly for extended space missions. These innovations in microgravity-based cartilage tissue engineering represent significant progress in both space-based and terrestrial regenerative medicine applications.

Cell Adhesion

Microgravity significantly alters cell adhesion properties, impacting both cell-cell and cell-matrix interactions. The absence of gravitational forces reduces the mechanical cues normally experienced by cells, which in turn affects the expression and function of cellular adhesion molecules (CAMs) such as integrins, cadherins, selectins, and the immunoglobulin superfamily (Ig-SF). These changes can disrupt cytoskeletal organization and signal transduction pathways critical for cell adhesion, migration, and overall cellular function. Studies have shown that endothelial cells, immune cells, stem cells, and other cell types exhibit modified adhesion behaviors under microgravity, which can affect processes such as cell recognition, migration, and cytoskeletal rearrangement [42,43].

Cell Proliferation

Proliferation of cells under microgravity conditions has been observed to either increase or decrease depending on the cell type and culture conditions. For instance, MSCs cultured in microgravity show enhanced proliferation and maintain their stemness better than those cultured under normal gravity. This is attributed to the altered expression of pluripotency markers like OCT4, SOX2, and NANOG. Microgravity also affects the cell cycle, often resulting in a higher proportion of cells in the S-phase, which is indicative of DNA synthesis and cell proliferation​​ [43,44].

Cell Differentiation

The differentiation of stem cells in microgravity is also markedly different from that in normal gravity. Microgravity has been shown to enhance the differentiation of MSCs into specific lineages, such as neuronal cells, by modifying the cellular environment and gene expression profiles. For example, simulated microgravity promotes osteoclast activity while inhibiting osteogenic differentiation in MSCs, which involves the downregulation of critical transcription factors and changes in cytoskeletal dynamics​ [44].

Advanced Cell Culture Techniques

RWV bioreactors: These bioreactors create a low-shear, high-mass transfer environment that mimics microgravity, promoting the formation of three-dimensional cell aggregates and spheroids, enhancing nutrient and waste exchange, and supporting more natural cell interactions and tissue formation​ [42]​.

Magnetic levitation and clinostats: These tools simulate microgravity by counteracting gravitational forces, enabling cells to grow in three dimensions. Magnetic levitation, for instance, uses magnetic fields to levitate cells, creating a microgravity-like environment that promotes better cell organization and tissue development​ [42,44].

Microsphere cultures: Combining microgravity with microsphere-based cultures allows for the selective proliferation of highly functional cells. For example, human adipose-derived stem cells (hASCs) cultured with polystyrene or collagen microspheres in a stirred suspension under microgravity conditions exhibit enhanced stemness and differentiation potential​ [42]​.

These advancements in microgravity cell culture techniques have significant implications for regenerative medicine, particularly in improving the quality and functionality of engineered tissues for therapeutic applications, both on Earth and during long-term space missions.

Cellular responses and regenerative potential

**Figure 1:**
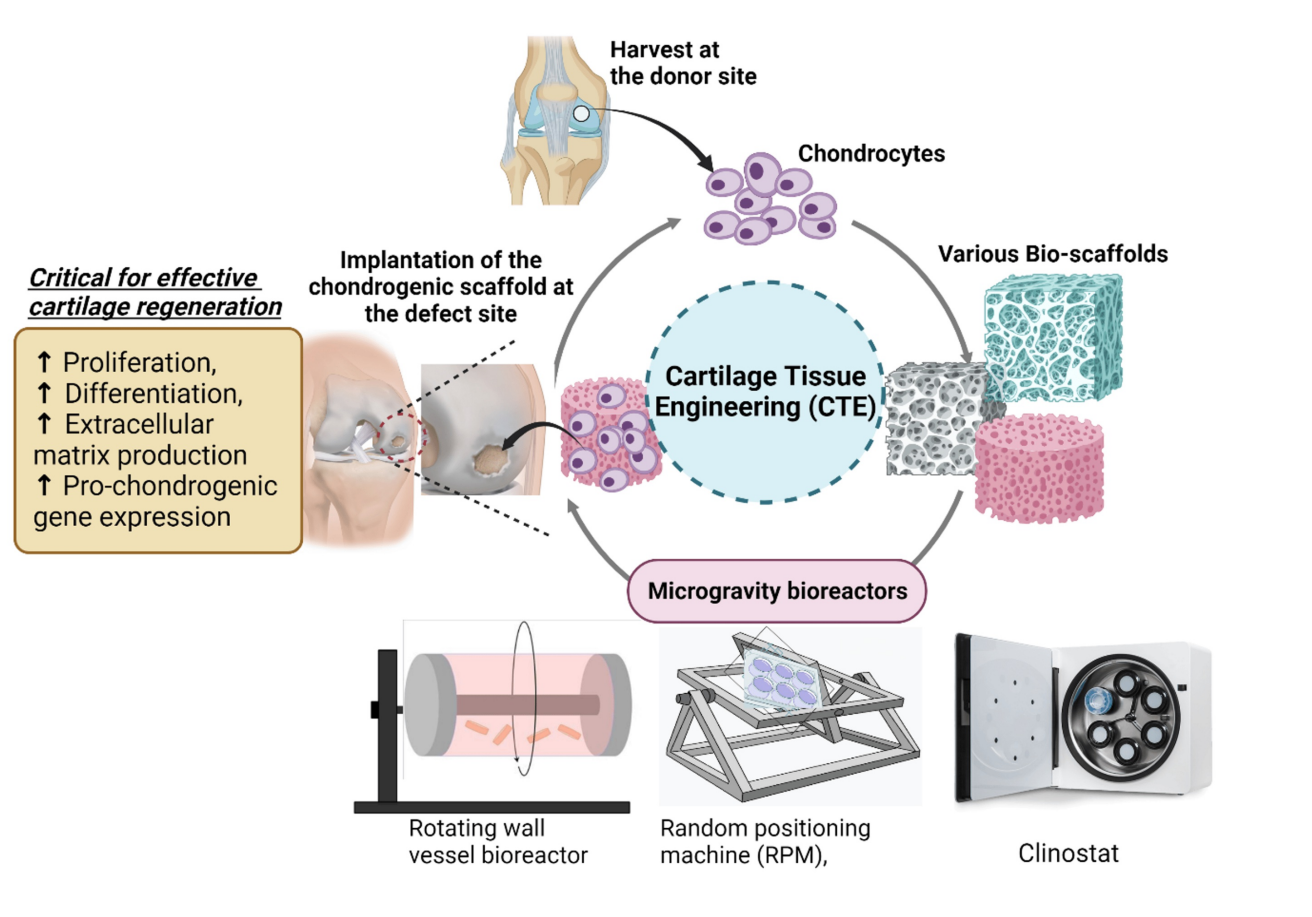
Strategies of CTE under microgravity CTE: Cartilage tissue engineering Picture courtesy: Dr. Madhan Jeyaraman and Dr. Sankalp Yadav

Cartilage cells, when exposed to microgravity conditions, demonstrate notable changes in their cellular behavior, including distinct alterations in gene expression and an increase in matrix synthesis. These modifications imply that microgravity might amplify the innate regenerative capabilities of cartilage, potentially leading to more efficacious therapies for cartilage repair and regeneration [2,3]. The strategies of CTE under microgravity are depicted in Figure 1. In microgravity environments, such cells display unique responses pivotal for tissue engineering. Altered gene expression, morphological changes, and different proliferation rates compared to Earth conditions have been observed. Research has shown that the SOX trio genes (SOX5, SOX6, and SOX9) are critically involved in chondrogenesis. In microgravity, these genes are often upregulated, which significantly enhances the chondrogenic differentiation of MSCs. SOX9, in particular, is a master transcription factor that regulates the expression of several downstream targets crucial for cartilage formation, such as collagen type II (COL2A1), aggrecan, and cartilage oligomeric matrix protein (COMP). The upregulation of these genes under microgravity conditions enhances the synthesis of ECM components, which are essential for the structural integrity and functionality of cartilage tissue​ [45,46]​. Furthermore, studies involving gene expression profiling have identified additional genes that exhibit altered expression in microgravity. For instance, connective tissue growth factor (CTGF) and collagen type I (COL1A1) are also affected. CTGF plays a role in the maturation of chondrocytes, and its modulation can influence the balance between proliferating and hypertrophic chondrocytes. The downregulation of osteogenic markers like COL1A1 in favor of chondrogenic markers suggests a shift towards enhanced cartilage formation and reduced ossification, which is beneficial for cartilage repair​ [46]​. These molecular changes underpin the improved regenerative properties observed in cartilage tissue engineered under microgravity. The enhanced ECM production and improved tissue architecture seen in these conditions suggest that microgravity can amplify the innate regenerative capabilities of cartilage, presenting new opportunities for therapeutic strategies in cartilage repair and regeneration​ [45]​. These variations reflect the adaptation of the cells to microgravity, potentially enhancing the regenerative potential of engineered cartilage tissue [1,47]. Furthermore, evidence suggests that cartilage tissue cultivated in microgravity possesses characteristics possibly superior to those developed under Earth's gravity. Enhanced ECM production and improved tissue architecture are among these features, essential for the functionality and longevity of the engineered cartilage. This makes microgravity a beneficial approach for creating high-quality cartilage tissue for clinical use [2]. The improved regenerative properties of cartilage tissue engineered in microgravity present new avenues for addressing cartilage-related injuries and degenerative diseases. Future research could concentrate on refining microgravity-based tissue engineering processes and investigating the practical application of this technology in clinical settings (Figure 1) [3].

Recent studies have placed significant focus on the cellular responses of cartilage cells in microgravity. These cells exhibit unique changes under such conditions, including alterations in cell morphology, gene expression, and protein synthesis, indicative of an enhanced capacity for regenerating cartilage tissue. For instance, it's been observed that chondrocytes in microgravity show an increased production of components vital for effective cartilage regeneration [5,48]. The potential of engineering cartilage tissue with improved biomechanical properties in microgravity could lead to more effective treatments for cartilage-related injuries and diseases [12]. Research into the cellular responses of cartilage cells under microgravity has unearthed key findings about their regenerative capacity. Microgravity seems to affect various aspects of cell biology, including cell morphology, proliferation rates, and differentiation pathways, which are crucial for the quality and functionality of the engineered cartilage. A notable observation in microgravity is the altered behavior of chondrocytes, the primary cells in cartilage. These cells display enhanced proliferation and an increased ability to produce extracellular matrix components, which are fundamental for robust cartilage formation. Furthermore, gene expression related to cartilage development and regeneration is also modulated in these conditions, underscoring the potential benefits of microgravity in CTE [1,5].

These cellular responses under microgravity not only shed light on the essential aspects of cartilage biology but also emphasize the improved regenerative capabilities of cartilage tissues developed in such environments. This knowledge holds significant promise for advancing treatments for cartilage-related injuries and diseases. The examination of cellular responses in microgravity conditions offers profound insights into the regenerative potential of cartilage tissue. This environment affects key biological processes such as cell morphology, proliferation, and differentiation, all of which are critical in the context of tissue engineering. In microgravity, cells show changes in gene expression and signaling pathways, leading to modified behaviors in stem cells and chondrocytes [4]. These changes enhance their capacity for cartilage formation. Despite the alterations, the unique microgravity environment supports the development of a more physiologically relevant ECM. This ECM is crucial for creating functional cartilage tissue, indicating that the modified gene expression and behaviors in microgravity ultimately contribute to improved tissue functionality. This enhances their capacity for cartilage formation. The unique microgravity environment aids in developing a more physiologically relevant ECM, vital for creating functional cartilage tissue. The regenerative potential of cartilage tissue cultivated in microgravity is indeed promising. Enhanced intercellular interactions and improved tissue structure suggest that microgravity can produce superior tissue compared to traditional methods. These findings have profound implications for regenerative medicine and space healthcare, providing innovative approaches for cartilage repair and regeneration both in space and on Earth.

Challenges and future directions

Microgravity presents a unique environment for the advancement of CTE, but it also brings forth a multitude of challenges that need to be addressed (Table 4).

**Table 4:** Challenges and future directions in microgravity-based CTE CTE: Cartilage tissue engineering

Challenges	Future directions
High costs and technical complexity	Develop cost-effective methods and technologies to simulate microgravity on Earth
Limited access to space missions	Increase collaboration between space agencies and biomedical researchers
Scalability and Earth-based application	Focus on scalable bioreactor designs and protocols for integration into the human body
Long-term viability and functionality	Investigate the prolonged effects of microgravity and ensure tissue functionality post-implantation
Translation of research findings	Establish a framework for applying space-based research to Earth-based clinical practices

The difficulties in conducting experiments in a space setting, compounded by the high costs and limited access to space missions, pose significant technical hurdles [1]. A crucial aspect to consider is the translation of the microgravity findings to Earth-based applications, particularly ensuring that the cartilage tissues engineered in this novel environment remain viable and functional upon implantation in the Earth's gravity [1,13]. Moreover, the scalability and cost-effectiveness of space-based research pose considerable challenges. It is essential to establish methods that are not only accessible but also economically viable to simulate microgravity conditions on Earth. This would allow for broader research and application of the techniques developed in space. Investigating the long-term viability and integration of microgravity-engineered tissues once reintroduced into Earth’s environment is of paramount importance [5].

The complexity of accurately replicating the specific microgravity conditions found in space and scaling up the production of cartilage tissues for clinical use are further obstacles. Ensuring the long-term viability and functionality of the engineered tissues is a significant concern that needs to be addressed [12]. Future research efforts should therefore be directed toward refining bioreactor designs for more effective tissue growth, understanding the prolonged effects of microgravity on cartilage cells, and establishing protocols for integrating engineered tissues into the human body. Collaborative efforts among space agencies, biomedical researchers, and clinicians are crucial for the translation of these research findings into practical medical applications [1,5].

Extended research in the potential implications of long-term microgravity exposure on cartilage tissue could yield valuable insights with dual applications: for chronic conditions on Earth and for long-duration space missions. Studying the prolonged effects of microgravity on cartilage tissue may reveal unique cellular and molecular adaptations that could inform novel treatments for chronic degenerative joint diseases such as osteoarthritis [49]. For instance, understanding how chondrocytes maintain their phenotype and function in microgravity over extended periods could lead to new strategies for preserving cartilage health in patients with chronic conditions [50,51]. Moreover, this research is crucial for supporting future long-duration space missions, including potential Mars expeditions. As astronauts spend increasingly longer periods in space, understanding and mitigating the effects of microgravity on cartilage tissue becomes essential for maintaining crew health [52,53]. Future studies should focus on developing longitudinal experiments both in space and using Earth-based microgravity simulators to examine cartilage tissue changes over extended periods. This could include investigating alterations in ECM composition, cellular metabolism, and mechanical properties of cartilage tissue under prolonged microgravity conditions. Additionally, research into potential countermeasures, such as specialized exercise regimens or bioengineered scaffolds, could help maintain cartilage health during long-term space missions and potentially offer new therapeutic approaches for chronic joint conditions on Earth.

The potential for microgravity-based CTE is immense, with promising implications for regenerative medicine and the treatment of joint diseases. Continued research and development in this field could lead to significant advancements in treating cartilage-related injuries and diseases, potentially extending to other tissues and organs [3]. Addressing the aforementioned challenges and focusing on future research trajectories will be key to unlocking the full potential of this innovative field.

## Conclusions

CTE under space microgravity conditions holds significant promise for overcoming Earth-based methodological limitations. Microgravity fosters favorable cellular morphology, proliferation, differentiation, and ECM production, critical for developing superior cartilage tissues. However, a major challenge lies in ensuring the long-term viability and functionality of these tissues once reintroduced to Earth's gravity. Future research must focus on strategies to preserve the beneficial properties acquired in microgravity, such as gradual re-adaptation protocols and innovative preservation techniques. Addressing challenges like high costs, technical complexity, and scalability is crucial. Comparative studies and collaborative efforts across scientific and medical communities will be essential in translating microgravity CTE research into practical, Earth-based clinical applications. This approach not only promises advancements in treating cartilage-related injuries and diseases but may also benefit broader tissue engineering and regenerative medicine fields.
